# Exosome‐mediated miR‐4660 delivery inhibits OPN promoted hepatoma cells aggression through targeting LGALS3BP

**DOI:** 10.1002/ccs3.70073

**Published:** 2026-04-17

**Authors:** Cuihua Liu, Riwen An, Ting Lin, Lei Qu, Xiaopeng Zheng, Jingkun Lu, Mei Hong, Pengwei Zhao, Fangxin Zhao, Xuan Zhang

**Affiliations:** ^1^ College of Basic Medicine Inner Mongolia Medical University Hohhot Inner Mongolia China; ^2^ The Traditional Chinese and Mongolian Medicine Hospital of Hohhot Huhhot Inner Mongolia China

**Keywords:** exosome, hepatoma cell, invasion, LGALS3BP, migration, miR‐4660, OPN

## Abstract

Hepatocellular carcinoma (HCC) is an aggressive malignant tumor with poor prognosis due to its strong metastatic potential. Studies linked osteopontin (OPN) to increased HCC metastasis. However, the mechanisms, especially those involving exosomes, are not well elucidated. This study investigates how OPN influences HCC cells behavior via exosome mediation. A stable HCC cell line overexpressing OPN, named SMMC‐OPN, was established. Co‐culture experiment revealed that exosomes from SMMC‐OPN cells enhanced the migration and invasion of the parental SMMC‐7721 cells. RNA sequencing found the downregulation of miR‐4660 in these exosomes, which targets LGALS3BP (galectin‐3 binding protein), elevated in both SMMC‐OPN cells and their exosomes. Treatment with SMMC‐OPN exosomes resulted in an upregulation of LGALS3BP expression in SMMC‐7721 cells. Notably, upon forced overexpression of miR‐4660 in SMMC‐OPN cells, miR‐4660 was observed to be encapsulated in exosomes. Co‐culture experiments demonstrated that exosomes containing miR‐4660, derived from miR‐4660‐overexpressing SMMC‐OPN cells, counter‐acted the promigratory and invasive effects of SMMC‐OPN exosomes on recipient SMMC‐7721 cells. These findings suggest that the downregulation of miR‐4660 in SMMC‐OPN exosomes contributes to the enhanced metastatic potential of HCC through modulating LGALS3BP. Furthermore, miR‐4660 delivery via exosomes inhibits OPN‐promoted hepatoma cell aggression by targeting LGALS3BP, highlighting a potential therapeutic target against cancer metastasis.

## Graphical abstract

miR‐4660 was found to be downregulated in exosomes derived from SMMC‐OPN cells, a stable HCC cell line that overexpresses OPN. LGALS3BP has been identified as a direct target of miR‐4660, with its expression levels elevated in both SMMC‐OPN cells and their secreted exosomes. Exosomes from SMMC‐OPN cells promoted the migration and invasion of SMMC‐7721 cells by upregulating LGALS3BP expression in these recipient cells. Transfection of miR‐4660 mimics into SMMC‐OPN cells led to a decrease in LGALS3BP expression levels. Furthermore, exosomes derived from miR‐4660‐overexpressing SMMC‐OPN cells mitigated the pro‐migratory and invasive effects on recipient SMMC‐7721 cells by targeting LGALS3BP.

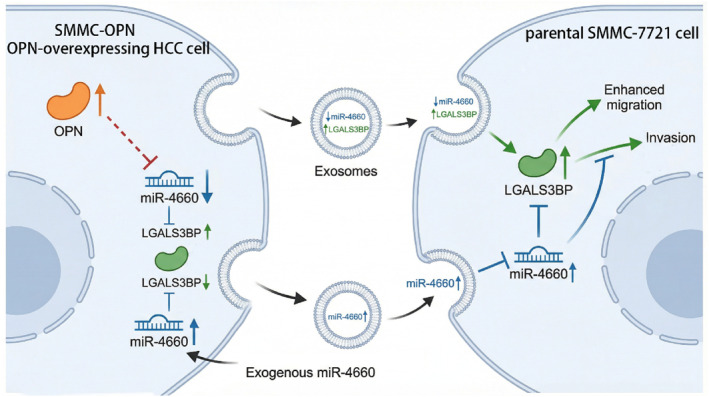

## 1 INTRODUCTION

Hepatocellular carcinoma (HCC) is the most frequent primary malignancy of the liver with a high malignancy and mortality rate. According to data, HCC ranks as the sixth most common cancer and the third leading cause of cancer‐related death in the world.^1^, ^2^ The high recurrence and metastasis rates are the main causes of the poor prognosis and mortality of HCC patients. Tumor metastasis is intricately linked to both the internal and external milieu of tumor cells, constituting a dynamic and complex process with multifactorial participation and multistep development that involves tumor cells themselves, tumor microenvironment interaction and other factors.^3^, ^4^, ^5^, ^6^ Therefore, in depth study in molecular mechanism underlying the HCC metastasis is of great significance for early diagnosis, postoperative treatment, and individualized therapy of HCC.

Osteopontin (OPN), known as secreted phosphoproteins (spp1), belongs to the small integrin binding ligand N‐linked glycoprotein family, which can be hydrolyzed by thrombin into amino‐terminal and carboxy‐terminal fragments.^7^, ^8^ The N‐terminal fragment specifically binds to integrins, whereas the C‐terminal fragment interacts with the adhesion molecule CD44. OPN plays a significant role in both physiological and pathological processes across various tissues and organs.^9^, ^10^, ^11^ Through its interactions with integrins and CD44, OPN regulates cell proliferation, adhesion, migration and invasion, thereby facilitating the development and metastasis in malignant tumors.^12^, ^13^, ^14^

Many documents indicate that OPN is upregulated and implicated in development of variety malignant tumors including liver, breast, brain, pancreatic, lung, and stomach cancer.^15^, ^16^ OPN enhances cancer cell survival, proliferation, migration, and invasion by interacting with various receptors and initiating a variety of cancer signaling pathways and cancer‐related factors.^15^, ^17^ Higher expression of OPN is involved in influencing glioma cell motility through the PI3K/AKT, JAK‐STAT, and syndecan 1 signaling pathways and relate to poor prognosis in glioma.^18^ In colorectal cancer liver metastasis, OPN drives immunotherapy resistance by stimulating CXCL12 production in cancer‐associated fibroblasts through activation of *β*‐catenin/HIF‐1α‐related transcription.^19^ In HCC, OPN promoted proliferation and migration by inducing JAK2/STAT3/NOX1 and reactive oxygen species production.^20^ Serum OPN demonstrates standalone diagnostic value and enhances conventional biomarker panels when combined with AFP and AST.^21^ Additionally, OPN is involved in tumor angiogenesis by inducing VEGF expression.^22^ In the tumor microenvironment (TME), OPN can promote tumor progression by recruitment immune cells and contributes in immunosuppression.^23^

Exosomes, extracellular vesicles with a diameter of approximately 30–150 nm, are participated in the regulation of numerous physiological and pathological processes, through transferring bioactive molecules, including proteins, lipids, mRNAs, and miRNAs between cells.^24^ Recent studies have underscored the significance of exosomes as a systemic, rapid and dynamic communication channel between intercellular communication in the TME contributing to cancer progression.^25^, ^26^, ^27^ miRNAs are endogenous non‐coding single‐stranded RNAs in eukaryotes, approximately 18–25 nucleotides in length, involved in post‐transcriptional regulation of gene expression.^28^ Because of its abundance in exosomes and regulatory functions, miRNA has attracted increased attention in recent years.^29^ The crosstalk between tumor cell and other cells in TME via exosomal miRNAs plays an important role in regulating multiple tumor metastasis and immunomodulation.^30^, ^31^, ^32^ However, it is unclear whether exosome miRNA is involved OPN induced liver cancer cell migration.

Our research found that exosomes derived from established OPN‐overexpressed HCC cell line could promoted receipt cells migration. In these exosomes, downregulation of miR‐4660 and upregulation of its target gene, LGALS3BP were observed. Subsequent investigation revealed that transfected miR‐4660 could be enclosed in exosomes and transferred into target cells to inhibit OPN‐mediated HCC cells migration and invasion by directly regulating LGALS3BP. These findings contribute to understanding the regulatory mechanism of OPN‐mediated HCC progression and identify a novel molecule in exosomes, miR‐4660, along with one of its key target genes, LGALS3BP, highlighting potential therapeutic targets for HCC intervention.

## 2 MATERIALS AND METHODS

### 2.1 Cell culture

Human immortalized normal liver cells (THLE‐2) and human HCC cell lines (SMMC‐7721, SK‐HEP‐1, and HepG2) were cultured in RPMI‐1640 (Gibco) medium containing 10% fetal bovine serum (FBS) (Gibco). SMMC‐P (SMMC‐7721 stably transfected with pcDNA3.0 vector) and SMMC‐OPN (SMMC‐7721 stably transfected with pcDNA3 vector containing OPN gene) cells were cultured in RPMI‐1640 medium containing 10% FBS supplemented with 300 mg/mLG418. Cultures were incubated at 37°C in a humidified atmosphere with 5% CO_2_.

### 2.2 Exosomes isolation

To isolate exosomes, fresh RPMI 1640 containing 10% exosomes‐free FBS (SBI, USA) was added to cells at 80% confluence. After 48 h incubation, the cell culture media was collected and centrifuged at 2000 rpm for 10 min and 10,000 rpm for 30 min to eliminate cells and debris. Transfer the cell‐free media into a fresh 15–50 mL conical tube. Exosomes were extracted using exosome purification kit (NORGEN, Canada) according to the manufacturer's instructions. Briefly, add 25 μL of ExoC buffer and 200 μL of slurry E to 10 mL of the cell‐free media. Vortex the mixture for 10 s and let stand at room temperature for 5 min. Then centrifuge for 2 min at 2000 rpm and discard the supernatant. Resuspended the slurry pellet using 200 μL ExoR buffer and incubated at room temperature for 5 min. After incubation, centrifuged for 2 min at 500 rpm. Transfer the supernatant to a mini filter spin column assembled with an elution tube and centrifuge for 1 min at 6000 rpm. Collected the flowthrough which contains purified exosomes. Bicinchoninic acid (BCA) protein assay kit (CWBio) was used to quantify the protein concentrations in exosomes.

### 2.3 Electron microscopy analysis

Drop 10 μL of exosomes solution on the copper grid, incubate at room temperature for 10 min, wash with sterile distilled water, and absorb the excess liquid with filter paper; absorb 10 μL of 2% acetic acid peroxidized uranium on the copper for negative staining for 1 min, absorb the floating liquid with filter paper, and dry under an incandescent lamp for 2 min. Place the grid under a JEM‐1400 transmission electron microscope (JEOL) for observation and image at 80 kv.

### 2.4 Nanoparticle tracking analysis (NTA)

We measured the exosome particle size and concentration using NTA with ZetaView PMX 110 (Particle Metrix) and corresponding software ZetaView 8.04.02. Isolated exosome samples were appropriately diluted using 1X PBS buffer (Biological Industries) to measure the particle size and concentration. NTA measurement was recorded and analyzed at 11 positions. The ZetaView system was calibrated using 110 nm polystyrene particles. Temperature was maintained around 23°C and 37°C.

### 2.5 Western blot

Cells or exosomes were homogenized in RIPA lysis buffer (Biomed), and the protein lysates were normalized using BCA protein assay kit (Thermo Fisher Scientific). The protein lysates were separated by 8% or 10% SDS PAGE and transferred onto PVDF membranes, blocked, and incubated with the different primary antibodies described above followed by HRP‐conjugated secondary antibodies. The protein bands were visualized using an ECL detection kit (PerkinElmer Life Science). The membranes were blocked 5% (w/v) with nonfat milk in PBS‐0.1% Tween 20 (PBS‐T)/for 1 h at room temperature and then incubated with the following primary antibodies diluted in PBS‐T for 2 h at room temperature. Subsequently, membranes were washed with PBS‐T and incubated with peroxidase‐conjugated secondary antibodies diluted in PBS T at room temperature for 1 h. Proteins detection was performed using enhanced chemiluminescence solution.

### 2.6 RNA isolation and cDNA synthesis

Exosome RNA extraction was conducted using the exosome RNA extraction kit (NORGEN) according to manufacturer's instructions. Cellular total RNA was extracted using TRizol (Invitrogen) and processed according to manufacturer's instructions. RNA quality and concentration were assessed with the Agilent 2100 Bioanalyzer (Thermo Scientific). Single‐stranded cDNA for miRNAs and mRNA was generated from the harvested RNA using the transScript miRNA first‐stand cDNA synthesis kit (Transgene) and transScript mRNA first‐stand cDNA synthesis kit (TransScript miRNA first‐stand cDNA synthesis), respectively.

### 2.7 RNA sequencing (RNA‐seq)

Total RNA concentration and integrity were assessed using the RNA Nano 6000 Assay Kit of the Agilent Bioanalyzer 2100 system (Agilent Technologies). A total amount of 3 μg total RNA per sample was used as input material for the small RNA library. Sequencing libraries were generated using NEBNext multiplex small RNA library prep set for illumina (NEB) following manufacturer's recommendations. Amplified libraries were purified on an 8% polyacrylamide gel for size selection. The 140–160 bp bands corresponding to adapter‐ligated constructs derived from the 21–40 nucleotide RNA fragments were recovered and dissolved in 8 μL of DNA elution buffer. At last, library quality was assessed on the Agilent Bioanalyzer 2100 system using DNA high sensitivity chips. RNA sequencing was performed for exosomes obtained from the SMMC‐P and SMMC‐OPN cells. miRNA expression levels were estimated by TPM (transcript per million) through the following criteria. Normalized expression = mapped readcount/totalreads*1,000,000. Differential expression analysis of two groups was performed using the DESeq R package. The *p*‐values was adjusted using the Benjamini & Hochberg method. Corrected *p*‐value of 0.05 was set as the threshold for significantly differential expression by default.

### 2.8 The reverse transcription‐quantitative PCR (RT‐qPCR)

RT‐qPCR amplification was performed using the quantitative SYBR green PCR kit. GAPDH and U6 small nuclear RNA were used as an internal normalized reference, respectively. To examine the specificity of the RT‐qPCR, the dissociation curve analysis was performed after a completed PCR. Relative mRNA expression levels were calculated using the 2−ΔΔCt method and normalized to GAPDH. Relative miRNA expression levels were calculated using the 2−ΔΔCt method and normalized to U6. The primers used were listed in Table A1.

### 2.9 Cellular uptake assay

A PKH67 green fluorescent kit was used to label SMMC‐OPN cells derived exosomes as described by the manufacturer. Briefly, exosomes were incubated with 1 μL of PKH67 and 200 μL of diluent C for 5 min, and then 200 μL of FBS was added to terminate staining. After washing twice with 1 × PBS, exosomes were purified and resuspended in 1 × PBS. SMMC‐7721 cells were seeded into 6‐well plates at a density of 4 × 10^5^ cells/well and incubated overnight in an atmosphere of 5% CO_2_ at 37°C. Then, cells were incubated with PKH67 labeled exosomes for 24 h at 37 37°C and washed three times with 1 × PBS. DAPI was used to label the cell nucleus. The cellular uptake was observed by Cytation 5 Cell imaging multi‐mode detection system (Agilent).

### 2.10 miRNA transfection

Cells were seeded in 6‐well plates at a density of 4 × 10^5^ cells/well and incubated for 24 h. Then, cells at 50% confluence were transfected wtih miR‐4660 mimics (50 nM), miRNA negative control (miR‐NC) (a commercially available scrambled miRNA sequence) (50 nM) or miR‐4660 inhibitor (50 nM) using riboFECT EL transfection reagent (RiboBio, China). All oligonucleotides were synthesized and purified by RiboBio (Guangzhou, China). Cells were harvested 24 or 48 h post‐transfection for further analysis.

### 2.11 Dual‐luciferase reporter gene assay

The online miRNA prediction tool TargetScan (http://www.targetscan.org) was used to predict the targets of miR‐4660. The reporter vector pGL3‐control containing LGALS3BP 3′UTR wild type or pGL3‐LGALS3BP 3′UTR mutant were constructed. Wildtype or mutant reporter constructs were co‐transfected into HEK‐293T cells in 24‐well plates with 50 nM miR‐4660 mimic or 50 nM miR‐NC and renilla plasmid by using Lipofectamine3000 (Invitrogen, USA). The reporter gene activities were measured by a dual‐luciferase reporter assay system (Promega) according to the manufacturer's instructions. Firefly luciferase activity was normalized for transfection efficiency using the corresponding renilla luciferase activity.

### 2.12 siRNA transfection

Cells were seeded in 6‐well plates at a density of 4 × 10^5^ cells/well and incubated for 24 h. Then, cells at 50% confluence were transfected with siRNA negative control (si‐NC) (does not target any known genes) and siRNAs targeting to OPN and LGALS3BP at a final concentration of 50 nM using riboFECT EL transfection reagent (RiboBio, China). All siRNA oligonucleotides (Table A1) were synthesized and purified by RiboBio (Guangzhou, China). Cells were harvested 24 or 48 h post‐transfection for further analysis.

### 2.13 Wound healing assay

Cells were seeded into the 6‐well plates and scratched with 200 μL pipette tips when cell density reached 70%–80% confluence. Cells were cultured in a complete medium without FBS and observed by microscopy at specific time points. The migration distance was determined by the difference between wound widths and analyzed by Image J software.

### 2.14 Transwell assay

Transwell assay was performed in 24‐well transwell inserts (Corning) with or without precoated Matrigel (Corning). Briefly, cells (1 × 10^6^) in serum free medium were respectively seeded into the upper chamber for 24 h, whereas the lower chamber was added with a medium containing serum. Noninvasive cells were removed by wiping with cotton swabs. The invaded cells adhered to the lower surfaces of insert membranes were fixed with methanol and stained with 0.1% crystal violet solution. Cells in five random fields was counted under a light microscope and calculated by Image J software.

### 2.15 Statistics analysis

The software GraphPad Prism version 8.0 and Image J were used for the data statistical analysis and image processing. All experiments were repeated at least 3 times independently. All data were presented as means ± SD. Student's *t* test was used to compare data between two groups. *p* < 0.05 was considered significant. Asterisks indicate significant statistical differences (*p < 0.05, **p < 0.01, ***p < 0.001).

## 3 RESULTS

### 3.1 SMMC‐OPN cells display higher migration and invasion abilities

**FIGURE 1:**
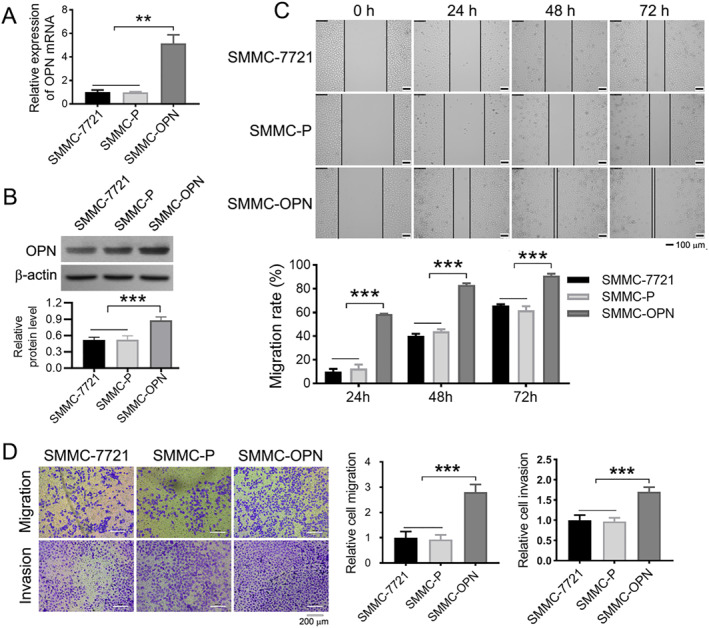
Higher migration and invasion abilities is present in SMMC‐OPN cells. (A) The expression level of OPN mRNA in SMMC‐OPN cells by RT‐qPCR. (B) The expression level of OPN protein in SMMC‐OPN cells by western blot. (C) Representative images of the wound healing assay. Scale bar: 100 μm. Histogram showed the statistical analysis of the cell migration rate. (D) Representative images of the Transwell assay. Scale bar: 200 μm. Histogram showed the statistical analysis of the relative migration and invasion of the cells. Data were presented as mean ± SD. *n* = 3, **p < 0.01, ***p < 0.001.

To confirm the promoting role of OPN in hepatoma cell aggressive behaviors, we firstly constructed SMMC‐OPN (SMMC‐7721 cells stably transfected with pcDNA3 vector containing OPN gene) and SMMC‐P (SMMC‐7721 cells stably transfected with pcDNA3 vector) cell lines. We showed that the expression of OPN in SMMC‐OPN was higher than that in SMMC‐7721 and SMMC‐P cells at both mRNA (Figure 1A, **p < 0.01) and protein (Figure 1B, ***p < 0.001) levels. Wound healing assay found that SMMC‐OPN cells exhibited a greater ability to repair the wound than SMMC‐7721 and SMMC‐P cells at 24, 48, and 72 h after wounding (Figure 1C, ***p < 0.001). Meanwhile, compared with SMMC‐7721 and SMMC‐P groups, more trans‐membraned cells were observed in SMMC‐OPN group by transwell assay (Figure 1D, ***p < 0.001). This data suggests that OPN strongly stimulates SMMC‐7721 hepatoma cell migration and invasion.

### 3.2 SMMC‐OPN derived exosomes enhance the migration and invasion of recipient cells

**FIGURE 2:**
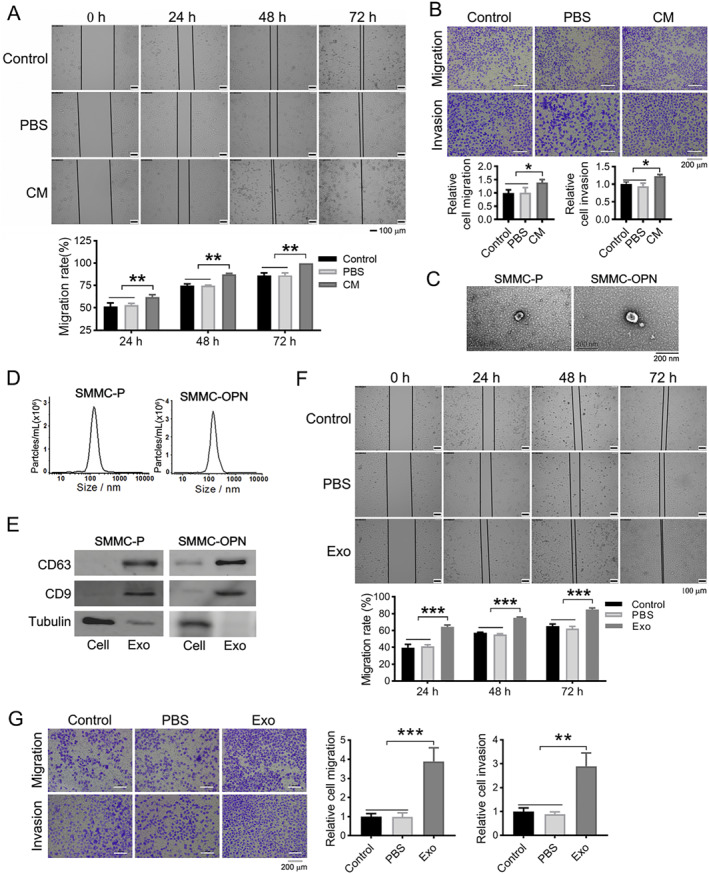
Exosomes from SMMC‐OPN cells contributed to enhanced aggression of recipient cells. (A, B) Cellular migration and invasion of SMMC‐7721 cells treated with conditioned medium of SMMC‐OPN: (A) Representative images of wound healing assay. Scale bar: 100 μm. Histogram showed the statistical analysis of cell migration rate; (B) representative images of the Transwell assay. Scale bar: 200 μm. Histogram showed the statistical analysis of the relative migration and invasion of the cells. (C–E) The characterization of exosomes derived from SMMC‐OPN cells: (C) Representative micrograph of exosomes by transmission electron microscopy, Scale bars: 200 nm; (D) the particle size of exosomes was determined by NTA; (E) western blot was conducted to detect the expression of exosome surface marker proteins. (F–G) Cellular migration and invasion of SMMC‐7721 cells after co‐cultured with exosomes (Exo) from SMMC‐OPN: (F) Representative images of wound healing assay. Scale bar: 100 μm. Histogram showed the statistical analysis of cell migration rate. (G) Representative images of the Transwell assay. Scale bar: 200 μm. Histogram showed the statistical analysis of the relative migration and invasion of the cells. Control: untreated recipient cells, PBS: treated with PBS buffer only (no exosomes). Data were presented as mean ± SD. *n* = 3, *p < 0.05, **p < 0.01, ***p < 0.001.

Given that exosomes mediate intercellular communication by shuttling between cells, the influence of exosomes from SMMC‐OPN cells on the aggressive behaviors of recipient cells was then explored. We firstly evaluated whether soluble factors from SMMC‐OPN cells could augment aggressiveness of SMMC‐7721 cells. Compared to un‐treating and PBS group, SMMC‐7721 cells treated with SMMC‐OPN cell conditioned medium displayed significantly higher ability of migration and invasion (Figure 2A,B, *p < 0.05, **p < 0.01). Next, exosomes secreted from SMMC‐OPN and SMMC‐P cells were isolated and analyzed. Under transmission electron microscopy, exosomes were observed to be a typical cup‐shaped morphology (Figure 2C). NTA of isolated exosomes revealed the size distribution with a peak diameter to be of approximately 140–150 nm (Figure 2D). The exosome specific markers CD63 and CD9^33^ were both positive according to Western blot (Figure 2E). SMMC‐7721 cells were then incubated with SMMC‐OPN exosomes. After incubation, SMMC‐7721 cells co‐cultured with exosomes exhibited a greater ability to repair the wound at 24, 48, and 72 h after wounding compared to control and PBS groups (Figure 2F, ***p < 0.001). Transwell migration and invasion assays indicated that more transmembraned cells were observed in SMMC‐7721 cells incubated with exosomes relative to that of control and PBS groups (Figure 2G, **p < 0.01, ***p < 0.001). These data suggested that exosomes were involved in the aggressiveness of OPN promoted hepatoma cells.

### 3.3 miR‐4660 is downregulated in exosomes secreted from SMMC‐OPN cells

**FIGURE 3:**
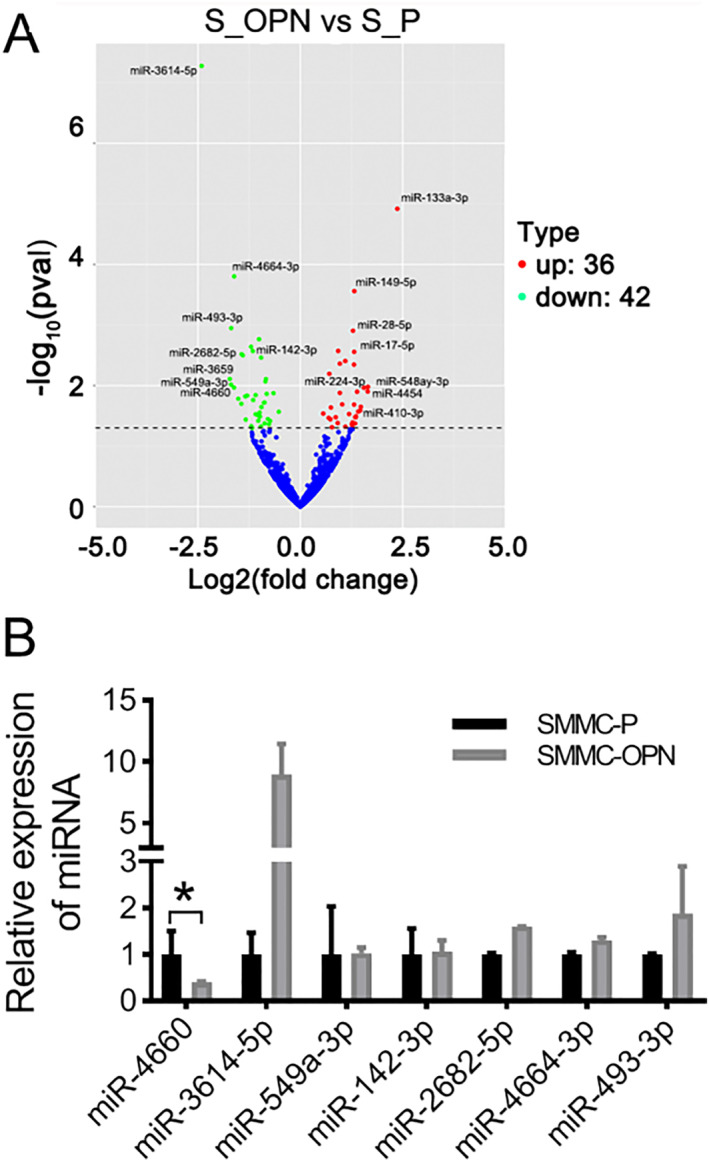
The expression of miR‐4660 was downregulated in SMMC‐OPN cell derived exosomes. (A) Differentially expressed miRNAs in exosomes derived from SMMC‐OPN and SMMC‐P cells using RNA‐seq. (B) The expression of downregulated miRNAs in SMMC‐OPN‐exo validated by RT‐qPCR. Data were presented as mean ± SD. *n* = 3, *p < 0.05.

To understand the key factors in exosome in affecting targeting cells, we extracted the total RNAs of SMMC‐P and SMMC‐OPN exosomes, and then profiled using RNA‐seq. According to the sequencing results, the expressions of 79 miRNAs were significantly altered in exosomes of SMMC‐OPN cells (36 up‐, 43 down‐) (Figure 3A). Consistent with the sequencing results, the RT‐qPCR validated that the expression of miR‐4660 was significantly down‐regulated in exosomes derived from SMMC‐OPN cells compare to SMMC‐P groups (Figure 3B, *p < 0.05).

### 3.4 miR‐4660 directly inhibit LGALS3BP expression by targeting its 3′UTR

**FIGURE 4:**
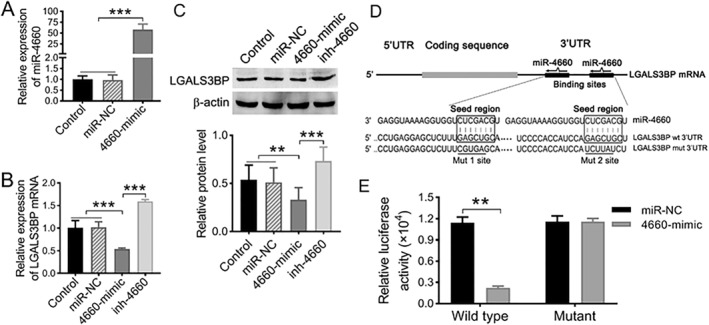
LGALS3BP was a target gene of miR‐4660. (A) The expression of miR‐4660 in SMMC‐7721 cells transfected with miRNA negative control (miR‐NC) and miR‐4660 mimics (4660‐mimic) were examined by RT‐qPCR. (B–C) The expression levels of LGALS3BP mRNA (B) and protein (C) in SMMC‐OPN cells transfected with miR‐NC, 4660‐mimic and 4660‐mimic together with miR‐4660 inhibitor (inh‐4660). (D) Predicted interactions between miR‐4660 and their two putative binding sites in the 3′UTR of LGALS3BP. Frame, seed match region; solid line, seed‐mutated region. (E) The effect of miR‐4660 on the activity of firefly luciferase reporter containing either wild type or mutant type 3′UTR was tested by dual‐luciferase reporter gene assay. Data were presented as mean ± SD. *n* = 3, **p < 0.01, ***p < 0.001.

To understand the downstream regulatory mechanism, we predicted and identified that LGALS3BP was one of potential target genes of miR‐4660 using TargetScan database.^34^ Analysis of data from the cancer genome atlas database using UALCAN online revealed that LGALS3BP expression is significantly upregulated in HCC tumors (Figure S1A, **p < 0.01). Meanwhile, results from RT‐qPCR displayed that the expression miR‐4660 is lower in SMMC‐OPN, SK‐HEP‐1, and HepG2 cells than in THLE‐2 cells (Figure S1B, **p < 0.01). The effect of miR‐4660 on regulating expression of LGALS3BP was subsequently examined via overexpressing miR‐4660 in SMMC‐7721 cells. A more than 50‐fold increase in the expression of miR‐4660 was observed in SMMC‐7721 cells transient transfected with 50 nM miR‐4660 mimics (termed 4660‐mimic) relative to the cells untransfection (Control) and transfected with 50 nM miR‐NC (termed miR‐NC) (Figure 4A, ***p < 0.001) Overexpression of miR‐4660 resulted in an obvious downregulation of LGALS3BP at both mRNA (Figure 4B, ***p < 0.001) and protein (Figure 4C, **p < 0.01, ***p < 0.001) levels. Meanwhile, co‐transfection of miR‐4660 inhibitor (inh‐4660) and 4660‐mimic was able to restore the expression of LGALS3BP (Figure 4B,C, **p < 0.01, ***p < 0.001).

To further validate whether LGALS3BP was the direct target gene of its miR‐4660, a dual‐luciferase reporter system was employed. According to bioinformatics analysis, there were two binding sites of miR‐4660 in the 3′‐untranslated regions (3′UTR) of LGALS3BP gene (Figure 4D). We cloned LGALS3BP 3′UTR sequences containing the predicted target sites (wild type) of miR‐4660 or dual‐site mutated sequences (mutant) into the pGL3 control vector, respectively. The data displayed that, compared with the miR‐NC group, co‐transfection of miR‐4660 mimics significantly decreased the relative firefly luciferase activity of the reporter with wild type 3′UTR (80.7%) but not that of the mutant reporter (Figure 4E, **p < 0.01), indicating that miR‐4660 can directly target the 3′UTR of LGALS3BP.

### 3.5 LGALS3BP in SMMC‐OPN cells could be delivered to recipient cells by exosomes

**FIGURE 5:**
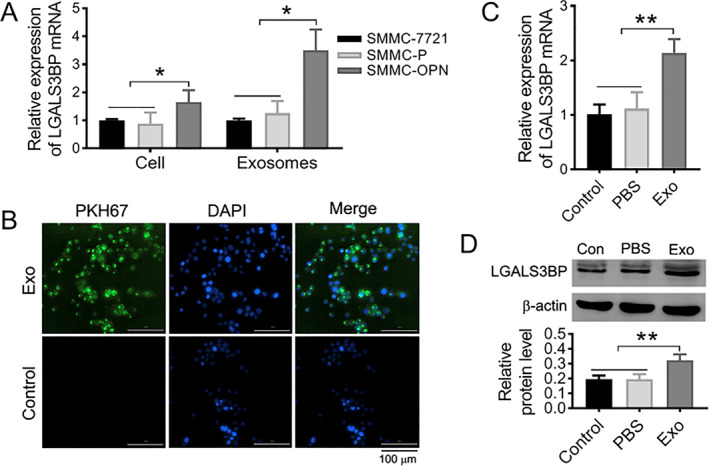
Increased LGALS3BP in SMMC‐OPN cells could be delivered to recipient cells by exosomes. (A) The expression of LGALS3BP mRNA in cells and exosomes detected by RT‐qPCR. (B) Fluorescence images of SMMC‐7721 cells treated with or without PKH67‐labeled SMMC‐OPN‐Exos (green). All nuclei were counterstained with DAPI (blue). Scale bar: 100 μm. (C, D) The expression levels of LGALS3BP mRNA (C) and protein (D) in SMMC‐7721 cells after 24 h of incubation with SMMC‐OPN derived exosomes. Control: untreated recipient cells, PBS: treated with PBS buffer only (no exosomes). Data were presented as mean ± SD. *n* = 3, *p < 0.05, **p < 0.01.

To further understand the role of LGALS3BP in OPN promoted HCC cell aggressive via exosomes, the background mRNA expression of LGALS3BP in SMMC‐OPN cells and exosomes was detected by RT‐qPCR. The results showed that compared with the SMMC‐7721 and SMMC‐P groups, the mRNA expression level of LGALS3BP gene was significantly higher in both SMMC‐OPN cells and their released exosomes (Figure 5A, *p < 0.05). To investigate the effect of OPN on the expression of miR‐4660 and LGALS3BP in various HCC cell lines, we assessed OPN expression in SMMC‐7721, SK‐HEP‐1, and HepG2 cells. The results indicated that endogenous OPN expression was highest in HepG2 cells (Figure S2A and S2B, **p < 0.01, ***p < 0.001). Following the knockdown of OPN in HepG2 cells, we evaluated the levels of LGALS3BP and miR‐4660. The findings revealed that, compared to the untreated (Control) group and the si‐NC group, the protein expression of LGALS3BP significantly decreased (Figure S2C, ***p < 0.001), whereas the expression of miR‐4660 increased in the siRNA targeting to OPN (si‐OPN) group (Figure S2D, **p < 0.01). Next, the exosomes isolated from SMMC‐OPN cells were labeled with PKH67 and then co‐cultured with SMMC‐7721 cells for 24 h. As shown in Figure 5B, SMMC‐7721 cells could effectively uptake the exosomes. Meanwhile, significantly increased expression of LGALS3BP was observed in SMMC‐7721 after cocultured with SMMC‐OPN exosomes but not in un‐treating or PBS treating cells (Figure 5C,D, **p < 0.01). These data demonstrated that SMMC‐OPN cells could transfer LGALS3BP into recipient SMMC‐7721 cells via exosomes to enhance HCC cell aggressiveness.

### 3.6 Overexpression of miR‐4660 suppressed SMMC‐OPN cells migration and invasion accompanied by LGALS3BP downregulation

**FIGURE 6:**
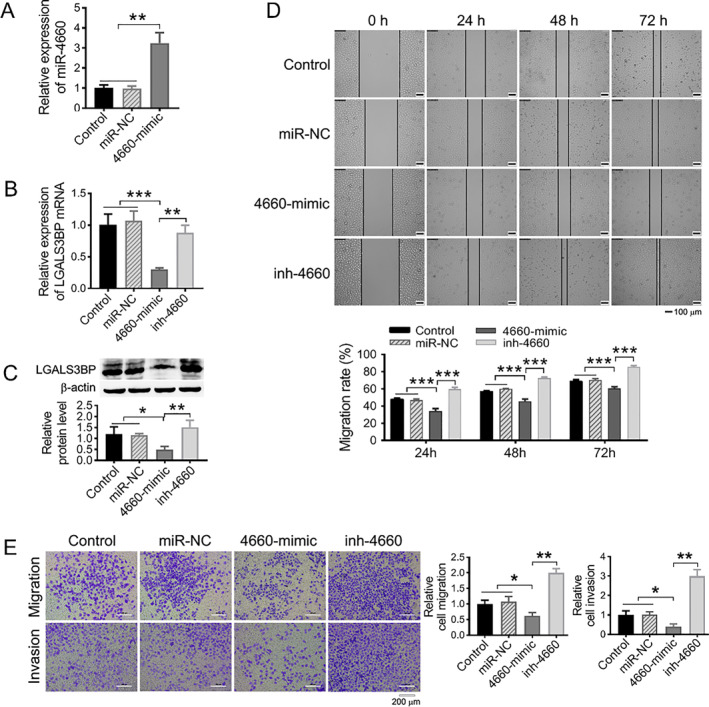
miR‐4660 inhibited SMMC‐OPN cell migration and invasion through targeting LGALS3BP. (A) The expression level of miR‐4660 were detected by RT‐qPCR in SMMC‐OPN cells transfected with miRNA negative control (miR‐NC) and 4660‐mimic. (B, C) The expression levels of LGALS3BP mRNA (B) and protein (C) in SMMC‐OPN cells transfected with miR‐NC, 4660‐mimic and 4660‐mimic together with inh‐4660. (D) The effect of miR‐4660 on SMMC‐OPN cell migration by wound healing assay (100 × scale). Histogram showed the statistical analysis of relative cell migration rate. (E) The effect of miR‐4660 on SMMC‐OPN cell migration and invasion by transwell assay. Scale bar: 200 μm. Histogram showed the statistical analysis of the migration and invasion of the cells. Data were presented as mean ± SD. *n* = 3, *p < 0.05, **p < 0.01, ***p < 0.001.

Subsequently, we investigated whether miR‐4660 could influence OPN promoted hepatoma cell aggressiveness through targeting LGALS3BP. miR‐NC, 4660‐mimic, and inh‐4660 together with 4660‐mimic were transfected into SMMC‐OPN cells, respectively, and then cell migration and invasion abilities were examined by wound healing assay and transwell assay. Overexpression of miR‐4660 was observable in 4660‐mimic group using RT‐qPCR detection (Figure 6A, **p < 0.01), suggesting that miR‐4660 was successfully transfected into SMMC‐OPN cells. Meanwhile, overexpression of miR‐4660 resulted in an obvious downregulation of LGALS3BP at both mRNA (Figure 6B, **p < 0.01, ***p < 0.001) and protein (Figure 6C, *p < 0.05, **p < 0.01) levels. Co‐transfection of miR‐4660 inhibitor (inh‐4660) and 4660‐mimic was able to restore the expression of LGALS3BP (Figure 6B,C, *p < 0.05, **p < 0.01). Wound healing and transwell assays showed that, compared with un‐transfected (control) and miR‐NC transfected groups, miR‐4660 overexpression significantly inhibited the migration and invasion of SMMC‐OPN cells, and this inhibition could be restored by co‐transfection of inh‐4660 (Figure 6D,E, *p < 0.05, **p < 0.01, and ***p < 0.001). To validate the functional role of LGALS3BP in these processes, we employed siRNA to knock down LGALS3BP in SMMC‐OPN cells. The results indicated that knockdown of LGALS3BP significantly inhibited the cell migration (Figure S3, ***p < 0.001). These data suggested that miR‐4660 could suppress hepatoma cell migration and invasion through targeting LGALS3BP.

### 3.7 miR‐4660 block the promotion of SMMC‐OPN derived exosomes on the recipient cells migration and invasion

**FIGURE 7:**
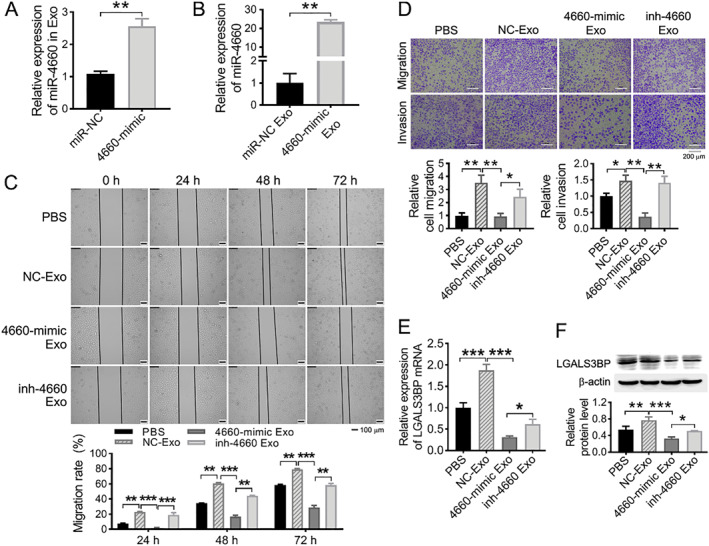
Exosome loaded miR‐4660 inhibits recipient cells aggression via LGALS3BP. Exosomes from miRNA negative control, 4660‐mimic and 4660‐mimic together with inh‐4660 transfected SMMC‐OPN cells (named NC‐exo, 4660‐mimic exo and inh‐4660 exo respectively) were incubated with SMMC‐7721 cells. (A) miR‐4660 expression in the above exosomes detected by RT‐qPCR. (B) miR‐4660 expression in SMMC‐7721 cells after 24 h of incubation with the exosomes. (C) SMMC‐7721 cells migration examined via wound healing assay after incubation with the exosomes for 24, 48 and 72 h (100 × scale). Histogram showed the statistical analysis of relative cell migration rate. (D) SMMC‐7721 cells migration and invasion tested via transwell assay after 48 h of incubation with the exosomes (100 × scale). Histogram showed the statistical analysis of cell numbers of migration and invasion. (E, F) The expression of LGALS3BP at mRNA (E) and protein (F) levels in SMMC‐7721 cells after co‐culture with the exosomes for 48 and 72 h was measured using RT‐qPCR and Western blot. *n* = 3, *p < 0.05、**p < 0.01, ***p < 0.001.

In order to explore whether miR‐4660 affect the promotion of SMMC‐OPN exosomes on the recipient cells migration and invasion, we isolated exosomes from SMMC‐OPN cells transfected with miR‐NC, 4660‐mimic, and 4660‐mimic together with inh‐4660, and named miR‐NC exo, 4660‐mimic exo, and inh‐4660 exo, respectively. RT‐qPCR detection showed that the miR‐4660 expression level was obviously higher in 4660‐mimic exo than that in miR‐NC exo (Figure 7A, **p < 0.01). Subsequently, SMMC‐7721 cells were treated with the above exosomes for 48 h, respectively. The miR‐4660 expression was increased in SMMC‐7721 cells treating with 4660‐mimic exo relative to the miR‐NC exo group (Figure 7B, **p < 0.01), indicating that miR‐4660 could be successfully encapsulated in exosomes and transferred into recipient cells.

Furthermore, wound healing and transwell assays were conducted to test the migration and invasion abilities of SMMC‐7721 cells after treating with the above exosomes. We found that SMMC‐7721 cells showed greater wound healing and transmembrane capabilities in miR‐NC exo treating groups compared to PBS groups. However, the promotion of SMMC‐7721 cells migration and invasion via miR‐NC exo treating were strongly abrogated in the 4660‐mimic exo treating group, and this abolishment could be rescued in the inh‐4660 exo treating group (Figure 7C,D, *p < 0.05, **p < 0.01, and ***p < 0.001).

Meanwhile, RT‐qPCR and Western blot results showed that the mRNA and protein expression of LGALS3BP in SMMC‐7721 cells were significantly up‐regulated after treating with miR‐NC exo relative to PBS group. The upregulated LGALS3BP expression in SMMC‐7721 cells treated with miR‐NC exo was dramatically abolished in the 4660‐mimic exo treating group, and this abolishment could be rescued in the inh‐4660 exo treating group (Figure 7E,F, *p < 0.05, **p < 0.01, and ***p < 0.001).

These results demonstrated that miR‐4660 could be delivered by exosomes and block the promotion of SMMC‐OPN derived exosomes on the recipient hepatoma cells migration and invasion by regulating the target gene LGALS3BP.

## 4 DISCUSSION

HCC is associated with a high mortality rate. Although radical resection significantly improves the survival time of HCC patients, their prognosis remains poor due to tumor invasion and metastasis. Consequently, exploring the metastatic mechanisms of HCC may aid in identifying strategies to inhibit its malignancy. Tumor metastasis is a dynamic and complex process involving multiple factors.^3^ Besides the ectopic expression of tumor metastasis‐related genes leading to modification in the inherent adhesion ability of tumor cells, various components in TME significantly influences the processes of tumor invasion and metastasis, including hypoxia, diverse cell lineages, signaling molecules, extracellular matrix components, and exosomes. Intercellular cross‐talk plays important roles in cancer progression and metastasis. Exosomes re‐leased by tumor cells have been proved to be effective cell‐to‐cell signal mediators. Previous studies established that OPN expression could be modulated through exosomes mediated cross talk between cancer cells.^35^, ^36^, ^37^ Although the role of OPN as a known metastasis‐related gene in the progression and metastasis of various cancers has been well documented.^38^, ^39^, ^40^ However, whether OPN could enhance HCC cells mobility and invasiveness by exosomes has not been reported.

To explore the functional roles of exosomes in OPN‐promoted HCC cells invasiveness, SMMC‐OPN, a highly metastatic HCC cell line, was established through stably overexpressing of OPN in low‐metastatic SMMC‐7721 cells. We showed that treatment with exosomes extracted from SMMC‐OPN cells significantly enhanced the migratory and invasive capacities of SMMC‐7721 cells, suggesting that OPN not only augments the migratory potential of hepatoma cells but also facilitates the secretion of exosomes that promote the migration of target cells. Additionally, according to Figure 2D, the exosome concentration in the SMMC‐OPN samples is actually higher than that in the SMMC‐P samples, suggesting that overexpression of OPN may increase the number of exosomes. All these findings mean that exosomes are involved in OPN‐mediated communication between high and low metastatic HCC cells. Exosomes modulate the activities of target cells through various signaling molecules they carry such as proteins and nucleic acids.^41^ Among these miRNAs are a class of important and very abundant signaling molecules in exosomes.^29^ In TME, tumor cells can influence adjacent cells via exosomal miRNAs, facilitating immune evasion and promoting tumor malignancy.^31^ Thus, further research focused on identifying the signaling molecules within exosomes that regulate the migration of SMMC‐7721 cells.

RNA‐seq was employed to analyze the ectopically expressed miRNAs in SMMC‐OPN exosomes. Notably, miR‐4660 was found to be significantly down‐regulated indicating a potential mechanism through which OPN may exert its effects. miR‐4660, an initially found novel miRNA, may be involved in the regulation of ovarian function, and^42^ can down‐regulate the expression of glyoxylate aminotransferase, which may be a biomarker of idiopathic oxalosis.^43^ Recent report showed that miR‐4660 was downregulated in bone marrow mesenchymal stem cells (BMSCs) without differentiation but increased during osteogenic differentiation.^44^ Interestingly, in osteosarcoma^45^ and triple negative breast cancer (TNBC),^46^ forced miR‐4660 overexpression could inhibit cancer progression, suggesting that miR‐4660 may be a new tumor suppressor. However, its role in HCC has not been reported. Our results indicates that the downregulation of miR‐4660 in exosomes of SMMC‐OPN cells may be responsible for the enhanced OPN‐mediated migration and invasion of hepatoma cells.

miRNAs exert their effects through the regulation on their target genes, so we next set out to identify the target genes of miR‐4660. Actually, we screened out 11 of potential target genes associated with cell adhesion and migration using on‐line analysis tools, including miRanda, Targetscan and miRDB. Notably, the mRNA expression level of LGALS3BP was found to be up‐regulated in SMMC‐OPN cells and their exosomes (Figure 5A), which correlated to the downregulation of miR‐4660 in above. Furthermore, Western blot and dual luciferase reporter gene assays confirmed that the expression of LGALS3BP was negatively regulated by the forced miR‐4660 overexpression in hepatoma cells.

Galectin‐3 binding protein (LGALS3BP), also referred to as Gal3‐BP, 90K, CyCAP, and Mac2‐BP, is a secreted highly glycosylated protein. High expression of LGALS3BP in serum is negatively correlated with the clinical prognosis of patients with various cancers (such as oral squamous cell carcinoma, lung cancer, liver cancer, and breast cancer),^47^ regulating tumorigenesis, cell adhesion, and immune evasion via interactions of cell‐cell or cells and the extracellular matrix. Depletion of LGALS3BP can effectively inhibit the metastasis of pancreatic ductal adenocarcinoma^48^ and hinder hepatocarcinogenesis by controlling the TGF‐β1 signaling pathway^49^ making it an important tumor biomarker. In this study, we test the contribution of LGALS3BP in OPN mediated hepatoma cells migration and invasion. The elevated levels of LGALS3BP in both SMMC‐OPN cells and their exosomes imply that OPN may modulate the expression of LGALS3BP through the suppression of miR‐4660. This notion is further supported by the observed increase in LGALS3BP expression in target cells co‐cultured with exosomes from SMMC‐OPN cells. This relationship underscores a potential regulatory axis in which OPN enhances LGALS3BP expression, thereby facilitating cell migration and invasion. Given that the depletion of LGALS3BP has been demonstrated to partially inhibit liver cancer development by modulating TGF‐β1 signaling,^49^ TGF‐β1 may serve as a potential downstream component of the OPN‐exosome‐miR‐4660‐LGALS3BP signaling axis. However, current research has not yet provided direct experimental evidence that connects the TGF‐β1 signaling pathway to this regulatory axis. This relationship requires further validation through experimental studies in the future. Additionally, this indicates that LGALS3BP may serve as a promising therapeutic target in HCC.

Given that miR‐4660 negatively regulates the expression of LGALS3BP, the inhibition of LGALS3BP through miR‐4660 may serve as a strategy to counteract OPN mediated hepatoma cells migration and invasion. Notably, we found that the forced overexpression of miR‐4660 in SMMC‐OPN cells resulted in reduced cell migration and invasion, indicating its potential role in suppressing HCC progression. This finding is consistent with previously reported functions of miR‐4660 in osteosarcoma and TNBC.^45^, ^46^ Interestingly, the overexpression of miR‐4660 was observed to be enclosed into the exosomes secreted by SMMC‐OPN cells and subsequently transferred to recipient cells. In the extracellular environment, free miRNAs are susceptible to hydrolyzation by RNase, which has limited the clinical application of miRNAs due to the safe, efficient and target delivery of miRNA. Exosomes, as naturally occurring lipid bilayer nanovesicles derived from cells, are characterized by low immunogenicity, high transport efficiency, good stability, and the capability to cross the blood‐brain barrier. These properties allow exosomes to effectively protect their contents from enzymatic degradation and immune system clearance, thereby prolonging their circulation half‐life and enhancing their biological activity, giving them significant advantages, and potential as drug delivery carriers.^50^ The above results suggest the therapeutic potential of miR‐4660 utilizing an exosome delivery system. Although the precise mechanism of miR‐4660 packaging and delivery into exosomes was not investigated in this study, we demonstrated that the migration and invasion of recipient cells, which were enhanced by SMMC‐OPN exosomes, were diminished by exosomes overexpressing miR‐4660 through regulating LGALS3BP. This highlight LGALS3BP as a potential therapeutic target, offering new insights into the molecular mechanisms underlying HCC progression.

Despite the promising results of this study in cellular model, several limitations remain that must be addressed. The findings of this study elucidate significant molecular mechanisms underlying the aggressive behaviors of hepatoma cells, particularly highlighting the role of OPN in the modulation of the expression of exosomal miR‐4660 and its target gene LGALS3BP, suggesting that OPN may influence the TME by altering the miRNA profiles of adjacent cells, thereby enhancing their migratory and invasive capabilities. However, the potential mechanism by which OPN regulates miR‐4660 expression is still unclear. Based on the existing literature, we propose that OPN may activate intracellular signaling pathways, such as PI3K/AKT and NF‐κB, upon binding to its receptors, including CD44 and integrins. This activation may subsequently regulate the promoter of the host gene miR‐4660, thereby influencing its transcription. Alternatively, OPN may modulate the activity of miR‐4660 processing enzymes, such as Drosha and Dicer, which could affect the levels of mature miR‐4660. Future studies should focus on exploring this precise upstream mechanism and the interactions among OPN, miR‐4660, LGALS3BP, and immune cell dynamics in HCC to fully elucidate the intricate relationships within the TME. Moreover, the notable upregulation of LGALS3BP in SMMC‐OPN cells and its consequent involvement in promoting cell migration and invasion highlight its potential as a biomarker for HCC progression. However, a deeper understanding of detailed molecular mechanisms, particularly the downstream signaling pathways regulated by LGALS3BP, is required. Whether other components in exosomes exert similar effects and participate in the mechanisms that drive changes in the hepatoma cells through alternative signaling pathways need further exploration. Additionally, the absence of in vivo validation restricts the ability to fully ascertain the physiological relevance of our findings within a complex biological context. Subsequent studies should verify in animal models whether exosome loaded miR‐4660 can exert a tumor suppressor effect in vivo. Nevertheless, this discovery provides a novel mechanism of OPN‐mediated HCC metastasis, which offers insights for identifying new targets for early detection and intervention. Targeting these mechanisms may represent a viable therapeutic strategy for HCC. Furthermore, it suggests the development of innovative interventions aimed at disrupting these signaling networks to mitigate cancer metastasis.

## 5 CONCLUSIONS

In summary, we identified a novel regulatory mechanism of OPN‐mediated HCC metastasis, characterized by the downregulation of miR‐4660 and the increased expression of LGALS3BP in exosomes, which enhances the migration and invasion of adjacent cells. The forced overexpression of miR‐4660 inhibits OPN‐induced aggression in hepatoma cells by targeting LGALS3BP through exosomal delivery, thereby exerting a tumor suppressor effect. The findings contribute to a deeper understanding of the molecular mechanisms underlying HCC progression and suggest pathways for future research and therapeutic development, providing insights for identifying new targets for the early detection and intervention of HCC.

## AUTHOR CONTRIBUTIONS

**Cuihua Liu**: Conceptualization, data curation, writing—original draft preparation. **Riwen An**: data curation, writing—review and editing, validation. **Ting Lin**: Writing—review and editing, software. **Lei Qu**: Writing—review and editing, data curation. **Xiaopeng Zheng**: Writing—review and editing, data curation. **Jingkun Lu**: Writing—review and editing, formal analysis. **Mei Hong**: Writing—review and editing, data curation. **Pengwei Zhao**: Writing—review and editing, visualization. **Fangxin Zhao**: Writing—original draft preparation, data curation, formal analysis, methodology. **Xuan Zhang**: Funding acquisition, project administration, supervision, writing—review and editing. All authors have read and agreed to the published version of the manuscript. Writing—original draft preparation.

## CONFLICT OF INTEREST STATEMENT

The authors declare no conflicts of interest.

## ETHICS STATEMENT

The Ethics Committee at Inner Mongolia Medical University approved this study.

## Supporting information

Supporting Information S1

## Data Availability

The datasets used or analyzed during this study are available from the corresponding author upon reasonable request.

## APPENDIX A

**TABLE A1:** Primers and oligonucleotides sequences.

Variable	Sequence (5′‐3′)
Primers	
OPN forward	ATCTCCTAGCCCCACAGAAT
OPN reverse	CATCAGACTGGTGAGAATCATC
LGALS3BP forward	AGGAACCCAAGGCGTGAAC
LGALS3BP reverse	TAGAAGATCTCCACGCGGC
GAPDH forward	ACAACTTTGGTATCGTGGAAGG
GAPDH reverse	GCCATCACGCCACAGTTTC
miR‐4660 forward	TGCAGCTCTGGTGGAAAATGGAG
miR‐3614‐5p forward	CCACTTGGATCTGAAGGCTGCCC
miR‐549a‐3p forward	TGACAACTATGGATGAGCTC
miR‐549a‐5p forward	AGCTCATCCATAGTTGTCACTG
miR‐142‐3p forward	TGTAGTGTTTCCTACTTTATGGA
miR‐2682‐5p forward	CAGGCAGTGACTGTTCAGACGTC
miR‐4664‐3p forward	TTCTTCCGGTCTGTGAGCCCCGTC
miR‐493‐3p forward	TGAAGGTCTACTGTGTGCCAGG
U6 forward	GCTTCGGCAGCACATATACTAAAAT
U6 reverse	CGCTTCACGAATTTGCGTGTCAT
siRNAs	
si‐OPN	GCCACAAGCAGTCCAGATT
si‐LGALS3BP	GGACCUGUAUGCCUAUGCATT
