# Supplementary material for: Exosome‐mediated miR‐4660 delivery inhibits OPN promoted hepatoma cells aggression through targeting LGALS3BP
Source: J Cell Commun Signal. 2026 Apr 17;20(2):e70073. doi: 10.1002/ccs3.70073 (PMC13090112; doi:10.1002/ccs3.70073)
Supplement: Supplementary file 1 — Supporting Information S1 [file CCS3-20-e70073-s001.docx]

**Supplements**


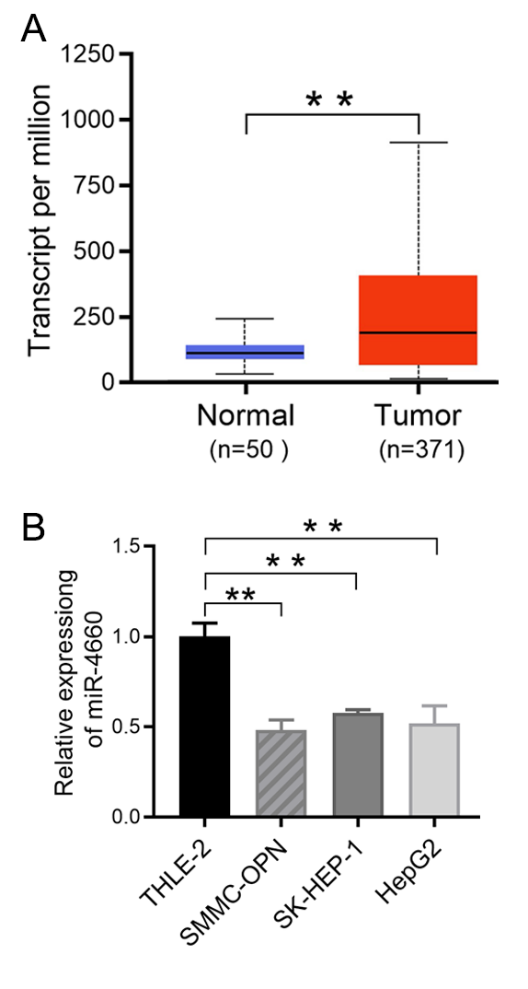


**Figure S1**: (A) Expression of LGALS3BP in liver hepatocellular carcinoma based on sample types. (B) Expression of miR-4660 in normal liver and HCC cells, Data were presented as mean ± SD, n=3, ***p* < 0.01.


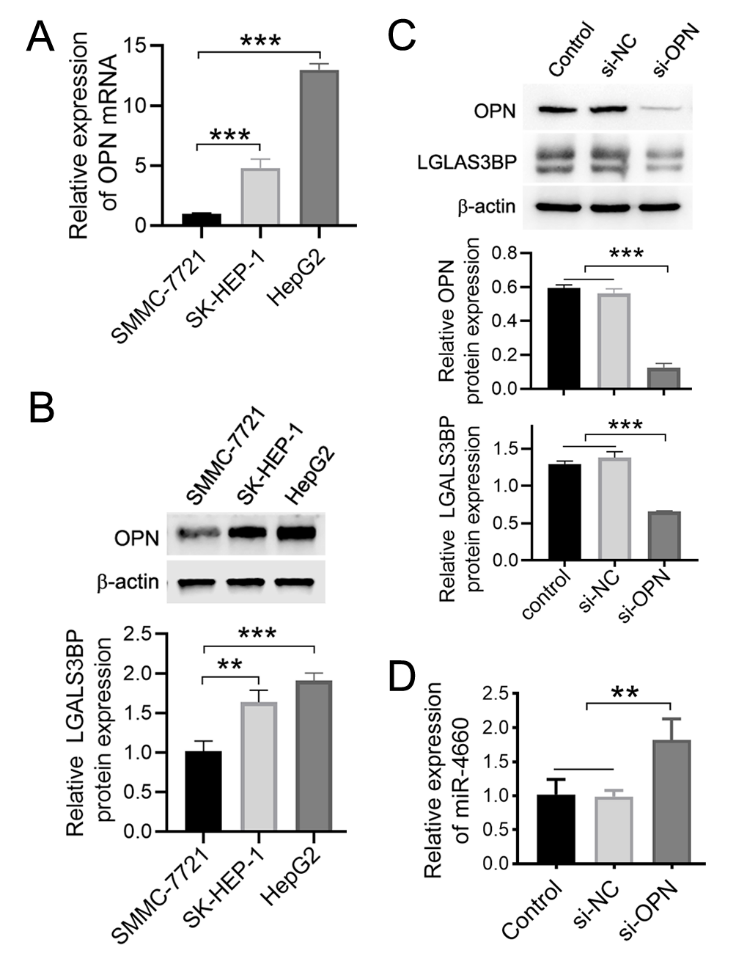


**Figure S2:** The effect of OPN on miR-4660 and LGALS3BP expression in HepG2 cells. (A-B) The expression of OPN mRNA (A) and protein (B) in 3 HCC cell lines. (C) The expression of LGALS3BP in HepG2 cells after knocking down OPN. (D) The expression of miR-4660 in HepG2 cells after knocking down OPN. Control: untreated cells, si-NC: siRNA negative control, si-OPN: siRNAs targeting to OPN. Data were presented as mean ± SD, n=3, ***p*<0.01, ****p*<0.001


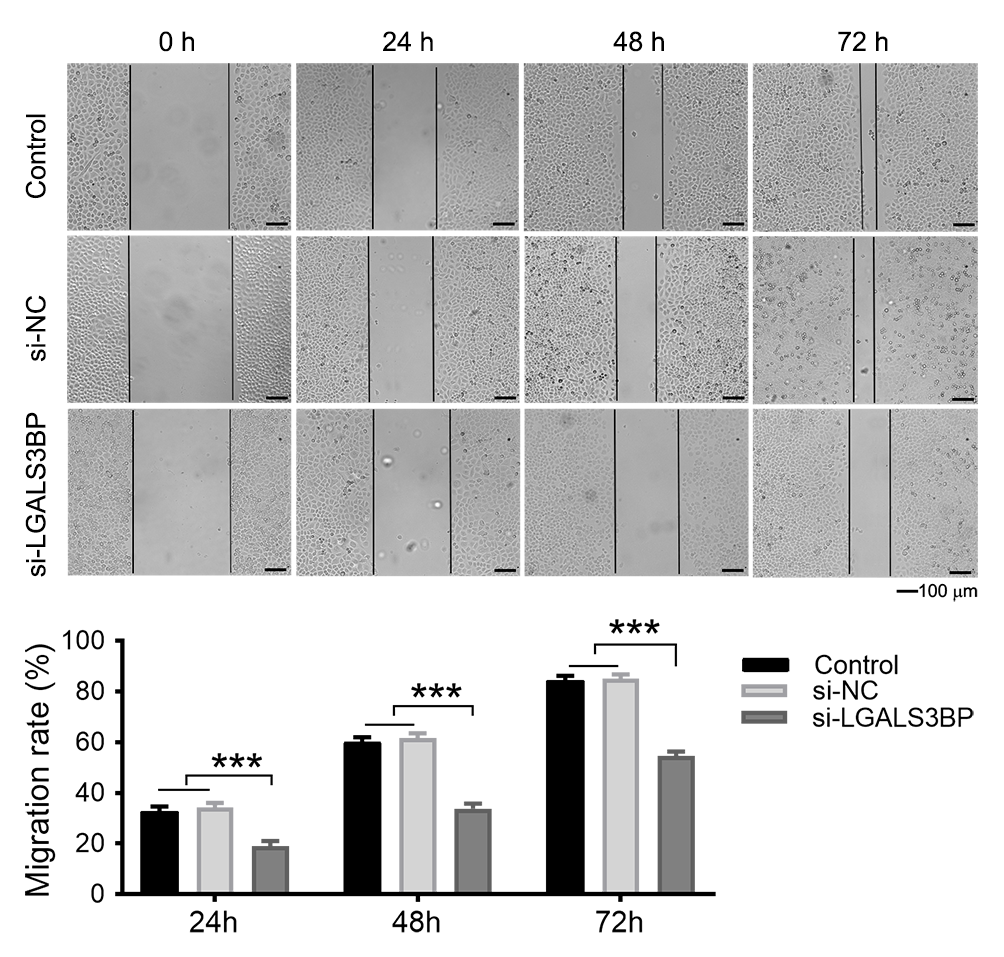


**Figure S3**: The migration of SMMC-OPN cells after knocking down LGALS3BP detection by wound healing assay (100×scale). Histogram showed the statistical analysis of relative cell migration rate. Control: untreated cells, si-NC: siRNA negative control, si-LGALS3BP: siRNAs targeting to LGALS3BP. Data were presented as mean ± SD, n=3, ****p* < 0.001.
